# Methodologies for Improving Antioxidant Properties and Absorption: 1st Edition

**DOI:** 10.3390/antiox14111290

**Published:** 2025-10-28

**Authors:** Daniela Tofani

**Affiliations:** Science Department, University of Roma Tre, 00146 Rome, Italy; daniela.tofani@roma3.it; Tel.: +39-0657333371

In recent decades, biological and biochemical research has greatly advanced our understanding of the significant effects of many natural antioxidants on various pathologies and diseases. Antioxidants have shown considerable potential not only in radical scavenging activity but also in reducing—and, in some cases, preventing—the development of degenerative diseases such as diabetes, Alzheimer’s disease, Parkinson’s disease, and several types of cancer.

Nevertheless, the practical application of natural antioxidants is still in its early stages, mainly due to intrinsic limitations such as low water solubility and absorption, short half-life, and rapid degradation in the gastrointestinal tract [1].

The Special Issue “Methodologies for Improving Antioxidant Properties and Absorption” aims to provide an overview of the most recent research in this field.

Various approaches to overcoming the drawbacks of natural antioxidants have been analyzed, including:

(i) The transformation of natural antioxidants into more stable and/or soluble derivatives;
(ii) The use of different types of delivery systems that can facilitate absorption and protect the molecule until it reaches its target cell or organ.

The first approach has led to the development of a new family of derivatized natural antioxidants, often obtained by combining two biological molecules to ensure the safety of the resulting derivatives. A common strategy involves glycosylation with simple sugars [2] or esterification with fatty acid [3], aimed at enhancing either water solubility or lipophilicity. Accordingly, many fatty acid esters of compounds such as quercetin [4] and hydroxytyrosol [5] have been synthesized and tested for their antioxidant activity and half-life, showing promising results.

In this context, Kim et al. [contribution 1] synthesized the ester of caffeic acid and hydroxytyrosol, which resulted in enhanced ROS scavenging capacity and a significant inhibition of melanin production in B16F10 melanoma cells with lower cytotoxic effect than that of caffeic acid.

More drastic transformations of natural antioxidants into more reactive derivatives can be carried out to enhance specific properties. Ionin et al. [contribute 2] oxidized the Siberian spruce galactoglucomannan–biopolymers, derived from softwood, obtaining an enhancement of heavy metal sorption capacity.

However, in the case of substantial modification of a natural antioxidant intended for pharmaceutical use, such as curcumin for anticancer therapy [6,7], the resulting molecules must be subject to the full range of toxicological evaluations required for the approval of a new drug.

On the other hand, the intense research conducted on delivery systems over the past few decades has found broad application in the field of antioxidants [8,9].

For instance, Mic et al. [contribution 7] used cyclodextrins to incapsulate a catechol hydrazinyl-thiazole derivative in order to reduce degradation and prolong the antioxidant effect over time.

Liposomes [10], solid lipid nanoparticles [11], emulsions, and nanoencapsulation techniques have been employed to enhance the skin absorption of antioxidant compounds in anti-aging cosmetics [contribution 8] as well as to improve the pharmacokinetic profiles of antioxidant drugs administered as adjuvants in various therapies for common age-related disorders, stroke treatment [12], and more severe diseases such as Alzheimer’s disease [13] and certain types of cancer [14].

Many studies aim to prolong the shelf life of antioxidants used as food preservatives by employing delivery systems [8,15]. Chen et al. used a Sm-cluster to enhance the performance of cysteine derivatives [contribution 5], while Merlino et el. investigated the antibacterial activity of an emulsion based on caper leaf essential oil [contribution 6].

A different approach to this problem involves the search for new natural derivatives that are more suitable for their intended applications. In this context, research focuses on agricultural and food industry wastes as potential sources of high-value antioxidants [16]. For instance, Poblete et al. analyzed the antioxidant and pro-inflammatory enzyme inhibitory activities of pisco grape pomace waste to identify bioactive molecules with potential nutraceutical applications [Contribution 3]. Similarly, Qamar et al. investigated the enzymatic pretreatment of industrial lignocellulosic waste derived from palm kernel meal to increase the concentration of antioxidant molecules and peptides, making it a valuable ingredient for use as a food supplement in both animal and human diets [Contribution 4].

Whatever the approach to the problem, it is increasingly evident that the full exploitation of antioxidant potential has yet to reach its peak in many areas of our lives—from food, nutraceuticals, and cosmetics to more specific pharmaceutical applications aimed at combating serious and degenerative diseases. Research in these fields is advancing rapidly, yet significant challenges remain before the full therapeutic and preventive potential of antioxidants can be realized. Nevertheless, each contribution adds to a more comprehensive understanding of antioxidant potential in modern science and technology.
